# Ecophysiological and Growth-Related Traits of Two Geophytes Three Years after the Fire Event in Grassland Steppe

**DOI:** 10.3390/plants11060734

**Published:** 2022-03-10

**Authors:** Zorica Popović, Vera Vidaković

**Affiliations:** Department of Ecology, Institute for Biological Research “Siniša Stanković”—National Institute of Republic of Serbia, University of Belgrade, Bulevar Despota Stefana 142, 11000 Belgrade, Serbia; vera.vidakovic@ibiss.bg.ac.rs

**Keywords:** spring ephemerals, *Crocus reticulatus*, *Iris pumila*, plant traits, grassland steppe, post-fire growth

## Abstract

Deliblato Sands is the single largest expanse of sand in Europe. It is the most fire-prone area of Serbia due to the absence of surface water, sandy soils, specific microclimate conditions, and vegetation composition. Post-fire regeneration is a long-term process that includes many aspects of vegetation regrowth and habitat recovery. In the third year following one of the disastrous fires, the growth dynamics of two geophyte species in unburned and burned sites were studied. During the growing season, burned and unburned populations of *Crocus reticulatus* Steven ex Adam and *Iris pumila* L. were assessed for growth parameters (biomass production, specific leaf area, leaf area index) and leaf-level ecophysiological traits (photosystem II efficiency, chlorophyll amount, relative water content). Species acclimated differently to changed abiotic and biotic site conditions after the fire event. *C. reticulatus* burned and unburned populations differed significantly in terms of flowering phenology and ecophysiological traits, whereas *I. pumila* burned and unburned populations differed significantly in terms of growth parameters. The findings support the assertion that geophytes are generally well adapted to environmental disturbances. Species, however, responded differently to fire-induced changes in the physicochemical and biotic environment, depending on their ecological requirements and adaptive capacity.

## 1. Introduction

Most European dry grasslands are seminatural ecosystems that have evolved in response to specific environmental and disturbance factors. These factors were usually coupled with long-term traditional land use ranging from agricultural intensification to arable field abandonment [1]. Along with climate, herbivores, and human impact, fire is one of the most important drivers of the formation and maintenance of European grassland communities [2]. However, research into the impact of fire on flora, fauna, and habitat structure is still limited [3,4,5].

The area of Deliblato Sands (Southern Banat) is the most fire-prone in Serbia, according to all indicators (number of fires, burned area, severity). This is a unique geomorphologic sand dune formation dominantly consisting of mixed forests (80%) and steppe (14%) surrounded by an area of cultivated grasslands. Because of its large, fixed dunes, which were bare until the early twentieth century and formed a desert-like landscape and a semiarid continental climate, the area is known as the “Desert of Europe” or the “European Sahara.” According to geomorphic observations, the sands were either deposited by the Danube River during Pleistocene climatic oscillations or were blown by košava following fluvial deposition and loess formation in the Holocene [6]. Specific microclimatic conditions, relief, sandy soils, the absence of surface watercourses, and vegetation composition all contribute to this area being extremely fire-prone, with severe fires occurring several times from the 1950s onward [7]. In addition to wildfires, agricultural practices are a very common cause of severe fires in Serbia [8]. Patch burning is commonly used in the management of steppe in Deliblato Sands, typically at the end of winter and early spring, when dead vegetation or standing hay from the previous year is removed, and subsequent vegetation regrowth is stimulated to appear earlier for grazing cattle [7]. Burning crop residues at the end of the harvesting season is another risk for fire spread in late summer and early autumn [7].

Generally, because of relatively limited fuel availability, grassland wildfires are comparatively small and have a relatively moderate impact on soil properties and surface processes [9]. Fire is considered to have a positive effect on mesic grasslands like tallgrass prairie, a smaller and more variable impact on grasslands with intermediate precipitation (600 mm per year), and a negative impact on more arid grasslands [10]. Recent evidence indicates that the effect of fire on productivity in semiarid grasslands is neutral to negative, rather than strongly negative, as previously assumed [11]. The fire effect on the soil system is influenced by a variety of factors, including fire severity, intensity, climate conditions, soil type, and topography [12]. These factors, when coupled with a biotic component (microbial community, grazing intensity), contribute to the dynamics of post-fire vegetation recovery. Following fire disturbances, geophyte assemblages form the early succession stage, contributing to ecosystem services such as soil improvement with organic matter and increased pollination activity [13]. The post-fire recovery processes of grassland plant communities are strongly related to species traits such as photosynthetic pathways and reproductive strategies (reviewed in [14]).

Geophytes are plants with buds on belowground organs (bulbs, corms, tubers, rhizomes) that allow them to remain dormant during adverse conditions and rapidly grow when favorable conditions return [15]. Although geophytes’ life strategy is primarily associated with unfavorable seasonal conditions, it provides them with highly successful adaptive features in the face of recurring fire regimes [16], and some species are characterized as fire-dependent or fire-induced [17,18,19]. There is a considerable body of knowledge on post-fire geophyte species richness, abundance, and flowering [20,21,22], redistribution of stored resources into reproduction [23,24], seed germination [25,26], and photosynthetic acclimation [27]. In general, total geophyte assemblages were found to be unaffected by fire events and to respond immediately with intense blooming, re-establishing pre-fire populations several years after the fire event [28].

Geophytes are among the plants that appear in the initial post-fire succession vegetation stage, thus contributing to soil improvement, habitat recovery, and vegetation restoration. The utilization of stored resources allows them to reproduce successfully in the first post-fire growing season. During flowering and fruiting, however, the bulbs and aboveground organs compete for photosynthetically fixed carbon sinks [29]. The replenishment of carbohydrate reserves is determined by photosynthetic rates in the coming growing season [30]. The majority of available literature has focused on geophytes’ reproductive strategies, with less emphasis on their photosynthetic capacity and growth patterns following fire events. Ecophysiological and growth-related traits are crucial to understanding the processes that structure ecosystems and the responses of ecosystems to global change, and they provide valuable information about plant and ecosystem productivity [31]. The objectives of this paper are to (a) characterize the ecophysiological traits (photosynthetic efficiency, chlorophyll content, leaf water content) and growth parameters (biomass production, leaf area index, specific leaf area) of two species, *Crocus reticulatus* Steven ex Adam and *Iris pumila* L., from burned and unburned sites, and (b) to determine whether there are differences in post-fire response between these species.

## 2. Results

Three years after the 2007 fire, the late-winter weather conditions in 2010 included relatively higher air temperatures in comparison to monthly average values (maximum 16 °C on 24 February, monthly average 2.6 °C), the monthly amount of precipitation was 61.33 mm (with a maximum in mid-February), and no snow cover on the ground [32]. The first field observation was that the plants of *C. reticulatus* from the burned site abundantly emerged and began flowering earlier than the population from the unburned site (emergence 24 February, flowering 1 March in the burned population and emergence 1 March, and flowering 7 March in the unburned population, respectively). In the case of *I. pumila*, the timing of emergence and flowering occurred in both populations at the same time (emergence 25 March, flowering 18 April).

**Figure 1:**
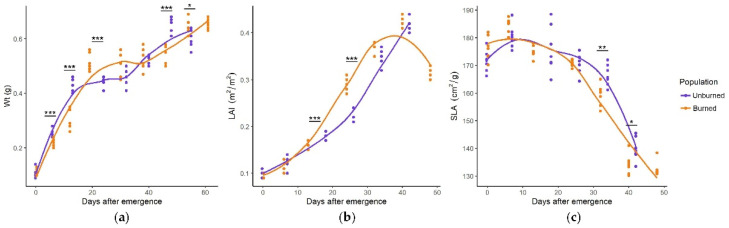
Seasonal dynamics of Wt (total plant weight, g) (**a**), LAI (leaf area index, m^2^/m^2^) (**b**), and SLA (specific leaf area, cm^2^/g) (**c**) in *C. reticulatus* plants from unburned and burned sites. Statistically significant differences are indicated with asterisks, * *p* < 0.05, ** *p* < 0.01, *** *p* < 0.001.

*C. reticulatus* plants from the burned and unburned site exhibited the same seasonal pattern of growth parameters (Figure 1a–c). Biomass increased rapidly within the first 20 days of emergence, followed by a plateau between days 20 and 40 before another general increase between days 40 and 60. These general tendencies were similar between burned and unburned populations of *C. reticulatus*, but the values for Wt significantly differed on five sampling dates. Total leaf area increased gradually with leaf age, peaking in the third part of the growing season, while SLA had maximum values in the first part of the growing season. Differences in SLA and LAI values were generally insignificant, except in a few sampling dates when LAI was higher (mid-season) and SLA lower (late season) in the plants from the burned site.

**Figure 2:**
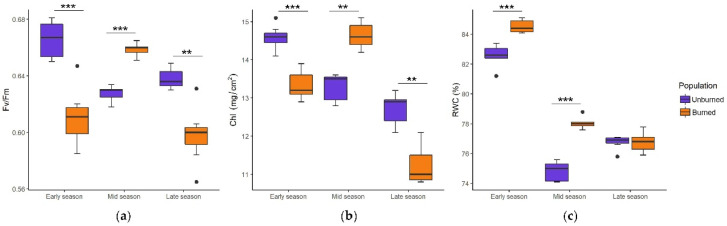
Seasonal dynamics of photosynthetic efficiency (Fv/Fm) (**a**), chlorophyll amount (Chl, mg/cm^2^) (**b**), and relative leaf water content (RWC, %) (**c**) in *C. reticulatus* plants from unburned and burned sites. Boxplots represent the median value (bar) within the interquartile range, IQR (colored rectangle), the range between minimum and maximum value that are at the maximal distance 1.5 × IQR from the first and third quartile, respectively (whiskers) and the outliers (black dots). Statistically significant differences are indicated with asterisks, ** *p* < 0.01, *** *p* < 0.001.

The values of ecophysiological traits and their seasonal tendencies in plants from burned and unburned sites differed significantly throughout the growing season (Figure 2a–c). Fv/Fm and Chl were at the highest level in the unburned population in the early season, but in the burned population, they reached a maximum in the mid-season. RWC showed a similar seasonal dynamic, although it was higher in the burned population.

**Figure 3:**
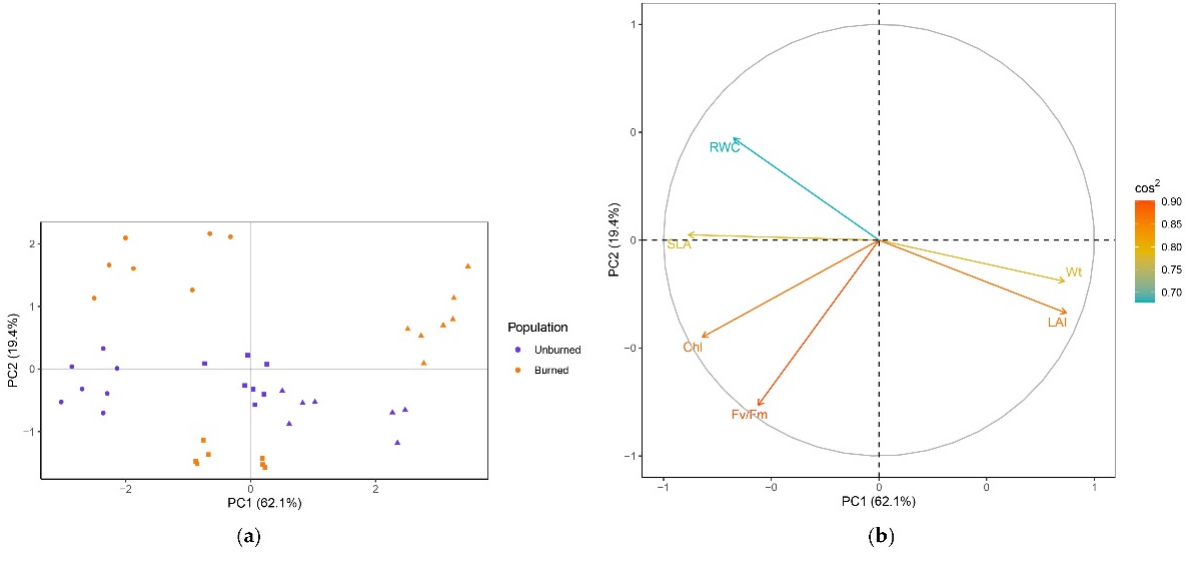
(**a**) PCA separation of *C. reticulatus* individuals in the plane of the first two axes. Individuals are colored by population (unburned site, population I, violet; burned site, population II, orange) and marked with a season symbol (early season, dot; mid-season, square; late season, triangle). (**b**) Loading plot with the structure of the six variables (plant traits) in the plane of the first two axes. The contribution of plant traits to the separation of individuals is indicated by the eigenvector scores (cos^2^).

PCA resulted in the first two principal components explaining 81.5% of the data variability (62.1% and 19.4%, respectively) (Figure 3). Based on the sum of squared cosines, all variables were well represented in the principal plane of the first two axes (0.68 < sum of cos^2^ < 0.90) (Figure 3b). Variables related to growth (Wt, LAI, SLA) had the most influence on the formation of PC1, while Fv/Fm was the major contributor to PC2. The clustering of individuals was clear in the plane of the first two PCs (Figure 3a). Individuals from the early season were more distinct, while those from the mid and late seasons partially overlapped. Early and mid-season individuals from both locations were segregated along PC1 by higher Chl, while higher Wt and LAI, and lower SLA contributed to the separation of late-season individuals. In the early season, plants from the unburned site were separated from those in burned site by lower RWC, while mid-season plants from the unburned site had lower Fv/Fm values compared with those from the burned site. The structure of the variables and their relations are visualized in Figure 3b. Wt and LAI were strongly positively correlated, while both were negatively correlated with SLA. LAI was also negatively correlated with RWC. There was no correlation between LAI and Fv/Fm.

**Figure 4:**
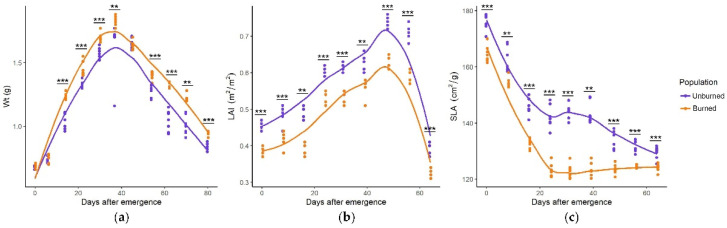
Seasonal dynamics of Wt (total plant weight, g) (**a**), LAI (leaf area index, m^2^/m^2^) (**b**), and SLA (specific leaf area, cm^2^/g) (**c**) in *I. pumila* plants from unburned and burned sites. Statistically significant differences are indicated with asterisks, ** *p* < 0.01, *** *p* < 0.001.

*I. pumila* plants from the burned and unburned sites significantly differed by growth parameters throughout the whole season. The seasonal pattern of growth parameters was similar, with maximum Wt in the middle of the growing season, while LAI gradually increased and SLA gradually decreased as the growing season progressed (Figure 4a–c). Values of all growth parameters differed significantly in nearly all sampling dates. Plants from the burned site had higher Wt, lower LAI, and lower SLA.

**Figure 5:**
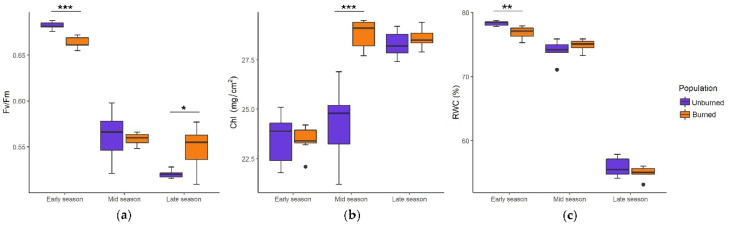
Seasonal dynamics of photosynthetic efficiency (Fv/Fm) (**a**), chlorophyll amount (Chl, mg/cm^2^) (**b**) and relative leaf water content (RWC, %) (**c**) in *I. pumila* plants from unburned and burned site. Boxplots represent the median value (bar) within the interquartile range, IQR (colored rectangle), the range between minimum and maximum value that are at the maximal distance 1.5 × IQR from the first and third quartile, respectively (whiskers) and the outliers (black dots). Statistically significant differences are indicated with asterisks, * *p* < 0.05, ** *p* < 0.01, *** *p* < 0.001.

The seasonal dynamics of ecophysiological traits (Fv/Fm, Chl, and RWC) were similar: Fv/Fm had the most favorable values at the start of the growing season, which decreased toward the mid and late seasons, whereas Chl gradually increased and RWC gradually decreased throughout the growing season. Depending on the sampling dates, differences in ecophysiological traits between populations were either significant or not significant (Figure 5a–c).

**Figure 6:**
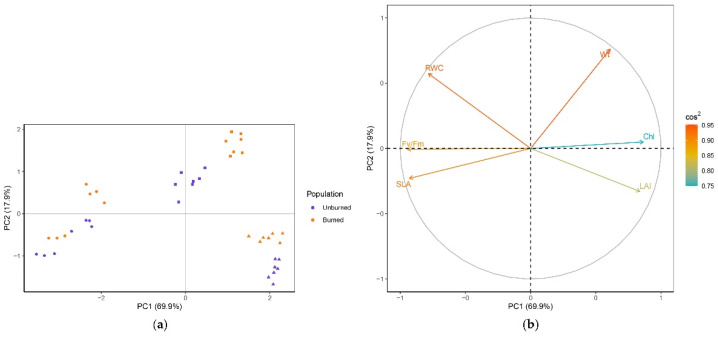
(**a**) PCA separation of *I. pumila* individuals in the plane of the first two axes. Individuals are colored by population (unburned site, population I, violet; burned site, population II, orange) and marked with a season symbol (early season, dot; mid-season, square; late season, triangle). (**b**) Loading plot with the structure of the six variables (plant traits) in the plane of the first two axes. The contribution of plant traits to the separation of individuals is indicated by the eigenvector scores (cos^2^).

The results from PCA showed that the first two axes explain 84.8% of the data variability (69.9% and 17.9%, respectively) (Figure 6). All variables were well represented in the plane of PC1 and PC2 (0.75 < sum of cos^2^ < 0.95). Fv/Fm, SLA, and Chl had the highest impact in the formation of PC1 and Wt in the formation of PC2. Individuals were clearly divided into early-, mid-, and late-season groups (Figure 6a). Early season individuals from both populations were separated by higher Fv/Fm and SLA, while mid-season individuals were separated by higher Wt and late-season individuals by lower RWC from other groups. In mid-season, plants from the unburned location were separated from those in th burned location by lower Wt, while late-season plants from the unburned site had higher LAI values compared with those from the burned site. The structure and relations of variables are shown in Figure 6b. Fv/Fm was positively correlated with SLA and RWC and negatively correlated with LAI and Chl. SLA was negatively correlated with chlorophyll and Wt, and LAI was negatively correlated with RWC. There was little or no correlation between Wt and RWC or LAI.

## 3. Discussion

The findings revealed different patterns of post-fire growth and ecophysiological traits in two geophyte species growing on sites with different fire histories. *C. reticulatus* plants in the burned site showed earlier emergence and flowering, as well as lower photosynthetic efficiency and leaf RWC than those in the unburned site throughout the whole season. In contrast, leaf-level ecophysiological traits in *I. pumila* plants in the burned site were not significantly affected. However, plants from the burned site displayed higher biomass production and lower values for SLA and LAI than plants in the unburned site. The fire occurred during the dormant season of geophyte species when foliage loss was irrelevant as a threat to survival, belowground resources were already filled, and buds were relatively protected below the soil [19]. Different patterns of acclimation to post-fire environmental quality between *C. reticulatus* and *I. pumila* in terms of growth parameters can be a mechanistic response to the disturbance and can be linked to species differences in storage organ type and ecological adaptations. It was reported that the growth and biomass partitioning of saffron (*Crocus sativus*) follows a pattern in which the biomass is primarily assigned to leaves and roots during the first stage of the vegetation cycle, while replacement corms receive the majority of the dry matter during the second stage [33]. The growth of replacement corms begins when the initial corm reserves have been mostly depleted and roots and leaves have reached their maximum size [34]. This process is dependent on photosynthetic rate, which is commonly limited under water stress. The unburned *C. reticulatus* plants had higher photosynthetic efficiency values throughout the whole season. This could imply that changes in certain site variables in the burned site were unfavorable to this species. Fv/Fm and Chl in plants from the burned and unburned sites showed different seasonal tendencies, whereas leaf RWC showed a similar seasonal decreasing trend. Plants from the unburned site had the highest Fv/Fm value at the beginning of the vegetation season when the leaf RWC was high. Unexpectedly, the plants from the burned site had the highest Fv/Fm value in the mid-season when the leaf RWC was low. This can be attributed to favorable temperatures (12.6 °C average in April) and better light availability due to the still incompletely developed surrounding vegetation in comparison with the unburned site.

According to some studies, the temperature near the ground and soil water content are triggering factors for flowering in *C. sativus* and *C. cambessedesii* [35,36]. These parameters may change in a burned area due to incomplete recovery of vegetation cover and ash and charcoal remnants. Low-to-moderate burn severity, which is common in grasslands, produces black ash and charcoal, which darkens the soil and affects its albedo and temperature [37]. The increase in soil temperature may favor the emergence of early ephemeral species [9] and affect the final dry mass, cell size, and leaf longevity [38]. Considering that the monthly maximum temperature (February 2010) in Deliblato Sands was recorded on 24 February (16 °C), it can be assumed that soil surface conditions contributed to better soil warming on the burned site and allowed for the earlier emergence of *C. reticulatus*. However, an experiment with controlled conditions would be required to gain a better understanding of these plant-soil relationships.

In terms of the duration of belowground organs, *C. reticulatus* corms last for 1 year, whereas *I. pumila* rhizomes last for approximately 4 years [39]. While *C. reticulatus* corms were completely replaced by new corms 3 years after the fire, it can be assumed that some >2 y old *I. pumila* rhizomes were still active at that time. Our results concerning growth parameters can be interpreted in the light of belowground organ preformation and resource allocation. Some studies suggest that belowground organ preformation in previous years affects the resource uptake and development in the current growing season and can limit environmentally induced changes in the growth and biomass allocation pattern [40]. The post-fire site conditions may have affected the formation of longer-lasting iris rhizomes, which was reflected in biomass productivity and resource allocation three years later. Lower leaf area parameters (LAI and SLA) values may indicate a greater allocation to reproductive organs in plants from the burned site, which is supported by maximum biomass production values during flowering and fruiting (4–6 weeks after emergence). However, this can only be confirmed with evidence from controlled experiments. It should also be noted that the flowering peak of *C. reticulatus* occurred on the 10th day after emergence, whereas the flowering peak of *I. pumila* occurred on the 20th day after emergence, and this phenological shift in flowering time may contribute to differences in dry matter partitioning between species [41].

Considering the species’ habitat response based on Elenberg values [42], *I. pumila* and *C. reticulatus* have similar ecological tolerances in terms of light conditions, moisture, and salt tolerance. However, there are differences in temperature ranges, with *C. reticulatus* being mesothermous and *I. pumila* being between a mesothermous and thermophilous species. In terms of nutrient and reaction indicator values, both species occur in more or less infertile areas and in moderately to weakly acid conditions. However, *I. pumila* has broader ecological adaptations toward more fertile soils and weakly basic to basic conditions. These general species’ adaptations are supported by our findings because post-fire soils in semiarid grasslands have increased N supply and pH [43,44,45,46,47,48], which are conditions that may be more favorable for *I. pumila* and contribute to higher biomass production in the burned site. Our study focused on phenotypic differences in plant traits between burned and unburned populations, with no genetic analysis, which would have provided more relevant information. The study with four grass species, however, revealed that growth-related differences were caused by developmental plasticity and prior fire exposure, and no consistent genetic differences were found between burned and unburned populations [49].

Plasticity in traits that enhance persistence in fire-prone ecosystems was documented in various growth forms, and evidence suggests phenotypic differences in ecophysiological and growth traits between plant populations from burned and unburned sites. Resprouting from belowground organs is a common regeneration mechanism in woody species after fire events, and plants that resprout have higher gas exchange rates and growth during the first season after a fire [50,51] due to the more available solar radiation and nutrients [52]. Fv/Fm values in *C. reticulatus* plants from burned and unburned sites differed significantly, with higher values recorded in the burned site in mid-season samples. This can be interpreted in terms of lower competitive pressure for resources (water, light) in the burned site where surrounding vegetation was not completely recovered. This is consistent with previous findings indicating that favorable water supply to leaves reduces the need for photoprotection while also contributing to increased photosynthetic efficiency [53]. Studies suggest that desert C4 grass species respond positively to fire by increasing net photosynthesis and stomatal conductance [54]. Increased photosynthetic rates in spring ephemeral *Zigadenus nuttallii* from the tallgrass prairie have been linked to increased nutrient supply in the post-fire environment, resulting in biomass compensation [27]. However, there have been reports that ecophysiological variables do not differ between plants with different fire histories and that photosynthetic rates are mostly sensitive to light level variation [55]. In late-season samples of *I. pumila*, higher Fv/Fm values were found in burned sites, and Fv/Fm was negatively correlated with Chl. Chlorophyll is a key indicator of plant photosynthetic capacity and physiological status, and it is closely related to N availability and the light environment [56]. The higher Chl throughout the whole season for *I. pumila* from the burned site can also be linked to the increased post-fire N content in the soil, as previously discussed. The study with six woody species found that chlorophyll content does not differ significantly between control and burned plants, implying that plant physiological stability can be restored even 6 months after burning [57]. In contrast to these findings, our study found significant variations in Chl content even 3 years after the fire, indicating that further research should reveal differences in this regard between woody and herbaceous species.

Spring ephemerals are a group of plants within the geophyte assemblage that commonly form specific vernal synisiae of the temperate forest understory or dry grassland gaps [58]. Despite their common ecological requirements, several studies confirmed subtle differences among coexisting species in terms of emergence, flowering time, photosynthetic performance, and biomass allocation to optimize the use of limited resources, specifically light before upper canopy closure [41,59,60,61,62,63]. The life span of dry grassland spring geophytes is limited by both predictable periodical environmental conditions (lack of light due to development of surrounding vegetation, lack of water due to beginning of dry season and presence of more competitors, overgrazing) and additional disturbances (management practices, fire events) [27,40,64,65,66]. This study contributes to our understanding of geophytes’ responses to fire disturbance on the level of ecophysiology and biomass production, and it demonstrates differences in their adjustments to post-fire environments that are related to species-specific ecological demands.

## 4. Materials and Methods

### 4.1. Site Description

Deliblato Sand is located in the Pannonian Plane’s south-eastern part (44°48′–45°1′ N, 38°36′–38°58′ E). It is Europe’s largest continental sand formation, with a semiarid continental Pannonian climate. The average annual air temperature is 10.2–11.8 °C, with the mean absolute maximum exceeding 30 °C, and the mean falling below −10 °C and big diurnal and annual fluctuations of temperature (−30 and +42 °C); the average annual precipitation ranges from 520 to 590 mm. Drought conditions prevail from July to October, with 1 month arid and 4 months semiarid. The plant communities *Corispermeto-Polygonetum arenariae* and *Festucetum vaginatae deliblaticum* represent the sandy type of vegetation, whereas *Koelerieto-Festucetum Wagnerii*, *Chrysopogonetum Pannonicum*, and *Festuco-Potentiletum arenariae* represent the steppe type. Geophytes account for 4.54% of the total number of species in the biological spectrum of the plant cover of Deliblato Sands [67]. *C. variegatus* and *I. pumila* are spring-flowering geophytes typical of Deliblato Sand grassland steppe communities, and their abundance cover values indicate that they are rare species [67]. Both species are protected by local and national regulations due to the threat of overharvesting of flowers, corms, and rhizomes.

The study was conducted on the southern part of Deliblato Sands (between the settlements of Dubovac and Kajtasovo), where the July 2007 large fire burned 333.50 ha of black and white pine plantations, 81.08 ha of deciduous species, 70.31 ha of shrub vegetation, and 61.90 ha of steppe vegetation. To monitor and compare the post-fire response of geophytes, two grassland sites (about 2 km apart), burned and unburned (each approximately 100 × 200 m), were chosen. It is difficult to state that two assessed locations were replicated grassland areas, one of which was exposed to fire. However, the formation of the community *Festucetum vaginatae deliblaticum* at the burned site began in the second post-fire year, indicating a high similarity with the unburned site where *Festucetum vaginatae deliblaticum* and *Koelerieto-Festucetum Wagnerii* communities were fragmentarily distributed. We compared differences in plant traits between populations of two geophyte species in two sites, one with fire incidence in the last few years and the other without recorded fire in the last few decades, assuming that a recent fire disturbance was the primary cause of the different site conditions. The soil in this area is black loamy sand, which has a higher clay and humus content than other types of sand and, thus, a relatively good ability to retain and store water; nitrogen content is 0.14–0.33%, and the reaction is slightly alkaline at pH = 7.51–8.61.

### 4.2. Sampling Procedure

The initial measurements of belowground organs were performed before the start of the vegetation season when the first leaves of crocuses (mid-February) and irises (early March) appeared. Not-emerged individuals’ belowground organs were excavated, washed from the ground, and weighed on a field balance (fresh weight, FW). About 200 belowground organs of average weight per species (~0.25 g in *C. reticulatus* and ~6.5 g in *I. pumila*) were sampled. These belowground organs were returned to their original position, covered with earth, the planting site was marked, and they served as a pool of specimens that were harvested periodically throughout the growing season. A small part of the underground organs (20 per population) was dried (in an oven at 75 °C until a constant dry weight), and the obtained dry weight values (DW: 0.08–0.1 g in *C. reticulatus* and 1.8–2.0 g in *I. pumila*) were used as the initial weight to monitor the growth dynamics. Individuals that emerged from these belowground organs were marked at the start of the growing season, used for in-field measurements, and harvested periodically to monitor the dynamics of biomass production and biometric variables (plant traits of 7–10 individuals per population were determined in each sampling).

### 4.3. Determination of Leaf-Level Ecophysiological Traits

Leaf-level ecophysiological traits (photosystem II efficiency Fv/Fm, chlorophyll amount Chl, and relative water content RWC) were assessed three times during the growing season (early, mid, and late season). All in-field ecophysiological measurements and leaf sampling for laboratory processing were carried out on sunny days between 9:00–11:00. Steady-state fluorescence was determined with a plant stress meter (BioMonitor S.C.I. AB, Umeå, Sweden) by the method of induced fluorometry [68,69]. The photosynthetic function (maximum efficiency of the photosystem II) was assessed by the rate of the basic fluorescence (Fv/Fm = (Fm − Fo)/Fm, where Fo and Fm are initial and maximal fluorescence of dark-adapted leaves). Each leaf was illuminated with saturating low light (100 µmol m^−2^ s^−1^) for 2 s, having spent at least 20 min in darkness. The chlorophyll content in the leaf tissue was determined spectrophotometrically, based on light absorption of a solution obtained after extraction with dimethyl sulfoxide (DMSO) [70]. One disk (1 cm diameter) per leaf was used to extract chlorophyll with 5 mL of DMSO. After incubation at 65 °C and until full extraction of chlorophyll was reached, the absorbance of each sample in 1.00 cm cuvettes was measured at 674.0 and 667.5 nm using a Shimadzu UV 160 spectrophotometer (Kyoto, Japan). The chlorophyll content was recalculated in relation to leaf area (mg/cm^2^) according to the methods used by Arnon [71]. Fully expanded leaves were measured for fresh weight (FW). Thereafter, leaves were water saturated until reaching a constant turgid weight (TW), measured, and oven dried at 75 °C until reaching a constant dry weight (DW). The relative leaf water content (RWC) was recalculated according to [72]: RWC (%) = ((FW/DW)/(TW/DW)) × 100.

### 4.4. Determination of Growth Parameters

Every 7–10 days, plants from each population were excavated with an undamaged root system. The plants were washed from the ground and dried at 75 °C until a constant dry weight was reached. Total dry mass of plant (Wt) was determined (sensitivity 0.1 mg, maximum load 5500 mg). For SLA determination, leaves were pressed between papers overnight and scanned the next day, and the area was calculated using digital image analysis. The leaves were then oven dried at 75 °C for 48 h and immediately weighed. The specific leaf area was obtained by dividing the one-sided area of a fresh leaf by its oven-dry mass [73]. To determine the leaf area index LAI, total leaf mass was sampled by a random sampling method of six squares (each 0.25 m × 0.25 m). All leaves from the sampling squares were scanned, and the total leaf area is shown in m^2^ of leaves per m^2^ of ground area [74].

### 4.5. Statistical Analyses

Populations from the burned and unburned sites for each species were compared on the basis of growth parameters (biomass, SLA, LAI) and ecophysiological traits (Fv/Fm, Chl, RWC). The assumption of normality for each variable in a given population was tested with the Shapiro-Wilk test before statistical analysis. A parametric test (two-sample *t*-test) was applied for comparing the populations when the variables appeared to follow a normal distribution (*p* > 0.05). A non-parametric test (two-sample Wilcoxon rank-sum test) was used when the variables did not appear to follow a normal distribution. Locally weighted scatterplot smoothing curve (LOESS) was used to graphically represent the seasonal variation in biomass, SLA, and LAI. The seasonal tendencies for ecophysiological traits (Fv/Fm, Chl/area, RWC) are represented in boxplot diagrams. Principal component analysis (PCA) was performed to visualize the data structure. All analyses were performed using R statistical software (version 3.4.0: The R Foundation for Statistical Computing, Vienna, Austria; URL https://www.r-project.org/ accessed on 21 April 2017).

## 5. Conclusions

The findings show that two geophyte species responded differently to fire disturbance. Biomass productivity increased in *I. pumila* plants on the burned site, where plants also had a smaller leaf area, resulting in thicker leaves. Higher Wt in mid-season samples and lower LAI in late-season samples in burned plants contributed the most to the separation of burned and unburned plants. Ecophysiological traits between sites had similar seasonal dynamics and mean values throughout the whole season and differed significantly only in some sampling dates. In contrast, *C. reticulatus* plants from two sites differed significantly in terms of ecophysiological traits, but differences in growth parameters were generally insignificant (SLA, LAI) or significant in some sampling dates (Wt). The differences in post-fire growth and ecophysiology between the two geophytes can be attributed to species’ ecological adaptations and the longevity of belowground organs. More information about species’ responses to individual and synergistic effects of fire-related environmental variables should be provided in future controlled experiments.

## Data Availability

The data presented in this study are available on request from the corresponding author.
